# Mechanistic Understanding of *Candida albicans* Biofilm Formation and Approaches for Its Inhibition

**DOI:** 10.3389/fmicb.2021.638609

**Published:** 2021-04-30

**Authors:** Tanu Atriwal, Kashish Azeem, Fohad Mabood Husain, Afzal Hussain, Muhammed Nadeem Khan, Mohamed F. Alajmi, Mohammad Abid

**Affiliations:** ^1^Medicinal Chemistry Laboratory, Department of Biosciences, Jamia Millia Islamia, New Delhi, India; ^2^Department of Food Science and Nutrition, College of Food and Agriculture Science, King Saud University, Riyadh, Saudi Arabia; ^3^Department of Pharmacognosy, College of Pharmacy, King Saud University, Riyadh, Saudi Arabia; ^4^Department of Tashreehul Badan, Faculty of Unani Medicine, Aligarh Muslim University, Aligarh, India

**Keywords:** *Candida albicans*, biofilm, resistance, antifungal drugs, prostaglandins, small molecule inhibitors, naturally occurring compounds

## Abstract

In recent years, the demand for novel antifungal therapies has increased several- folds due to its potential to treat severe biofilm-associated infections. Biofilms are made by the sessile microorganisms attached to the abiotic or biotic surfaces, enclosed in a matrix of exopolymeric substances. This results in new phenotypic characteristics and intrinsic resistance from both host immune response and antimicrobial drugs. *Candida albicans* biofilm is a complex association of hyphal cells that are associated with both abiotic and animal tissues. It is an invasive fungal infection and acts as an important virulent factor. The challenges linked with biofilm-associated diseases have urged scientists to uncover the factors responsible for the formation and maturation of biofilm. Several strategies have been developed that could be adopted to eradicate biofilm-associated infections. This article presents an overview of the role of *C. albicans* biofilm in its pathogenicity, challenges it poses and threats associated with its formation. Further, it discusses strategies that are currently available or under development targeting prostaglandins, quorum-sensing, changing surface properties of biomedical devices, natural scaffolds, and small molecule-based chemical approaches to combat the threat of *C. albicans* biofilm. This review also highlights the recent developments in finding ways to increase the penetration of drugs into the extracellular matrix of biofilm using different nanomaterials against *C. albicans*.

## Introduction

Biofilms are structurally organized microbial communities attached to the surfaces of implanted devices encapsulated within a solid protective extracellular matrix (Spormann et al., 2004). Approximately, 65% of all human microbial infections embroil biofilm formation. Therefore, identifying its role, composition, and impact of microbial biofilms on human medication is an attractive proposition (Douglas, 2003). Biofilm “inhabitants,” fungi and bacteria, are less sensitive or insensitive to antimicrobial agents. The property of the microorganisms to adhere to different surfaces facilitates the formation of biofilm in clinical settings such as catheters, prosthetic heart valves, joints, and various tissues in the host leading to effective colonization and this results in persistent drug-resistant.

In recent years, the prevalence of fungal infection has increased. These mycotic diseases account for over 10 lakhs of human deaths annually, which has become a delinquent health issue across the world. In today’s scenario, these opportunistic fungal infections have affected immunosuppressed or immunocompromised patients, and folks proclaimed for administration in the intensive care unit. These mycotic infections are classified from non-life-threatening mucocutaneous illnesses to intrusive infections which can affect any organ (Pfaller and Diekema, 2007; Yapar, 2014). This kind of fungal infection is often connected with the other mild to lethal fungal or bacterial infections involving species such as *Aspergillus* (Williams et al., 2016; Richardson and Rautemaa-Richardson, 2019) different species of *Candida*, *Staphylococcus aureus*, and *Pseudomonas aeruginosa* (Kaplan, 2014; Pleszczyńska et al., 2015; Fleming et al., 2017) and *Cryptococcus neofornams* (Aslanyan et al., 2017). Of the several known and frequently studied *Candida* species, *C. albicans* is the one most commonly recorded and encountered fungal pathogen in the human race (Su et al., 2018). Systemic candidiasis, caused by *C. albicans*, is known to be the reason for death in nosocomial and opportunistically abysmal fungal infection in patients (Su et al., 2018). followed by *Candida glabrata. Candida tropicalis* is commonly found in urinary tract infections (UTI) whereas *Candida parapsilosis* is mostly located on the epidermis of a healthy individual. The latter is also the causative agent of catheter-related infections. Likewise, all *Candida* species depict differences in terms of their biofilm formation, structure, changes in the morphology extracellular matrix (ECM), and potential to resist antifungal drugs (Cavalheiro and Teixeira, 2018).

National Nosocomial Infection Survey (NNIS) indicated that *Candida* spp. were the fourth most common cause of nosocomial bloodstream infections during the 1990s. However, in more recent studies, it has been discovered that *Candida* spp. are the third most frequent nosocomial bloodstream isolates (Perlroth et al., 2007; Lohse et al., 2018). Additionally, *Candida* is the third mainstream infection of central-line-associated bloodstream infections (CLABSIs) and the second most prominent reason for catheter-associated urinary tract infections in the United States. Lockhart (2014) Subsequently, the incidence of disseminated candidiasis which broadly refers to the mucosal, cutaneous, and deep-seated organ infection caused by *Candida* genus has increased 15 to 20-fold in the last two decades (Lockhart, 2014; Pappas et al., 2018). It has been reported that systemic candidiasis, caused by *C. albicans* is one of the major reasons for death in nosocomial as well as opportunistically abysmal fungal infection in patients because of its ability to form a biofilm which decreases the susceptibility of fungal cells toward drug treatment. Consequently, it has been reported each year that up to 50% of systemic candidiasis adults patients and approximately 30% of the young population die due to candidiasis co-related with biofilms. Moreover, there is an estimation of 100 thousand deaths because of the infection initiated by biofilm formation. Approximately, $6500 millions are spent per year in the United States toward its treatment causing a high fiscal impact on the state (Gulati and Nobile, 2016; Silva et al., 2017). The five most widespread species, *C. albicans*, *C. glabrata*, *C. parapsilosis*, *C. tropicalis*, and *C. krusei* are the reason behind 92 to 95% of Intensive Care Unit (ICU) cases in the United States, although, distribution of all five species might vary (Kojic and Darouiche, 2004; Ten Cate et al., 2009). According to National Institute of Health (NIH) reports, pathogenic biofilms account for more than 80% of all microbial infections (Ramage et al., 2006; Perlroth et al., 2007; Tsui et al., 2016; Lohse et al., 2018), of which 75% cases are of vaginal yeast infection in women once or more than once in their lifetime (Perlroth et al., 2007; Yapar, 2014).

*Candida albicans* is a commensal fungi, asymptomatically associated with normal microflora of the host, but becomes invasive and virulent when converted to hyphal form covered by an extracellular polymeric substance (EPS), which is a dimorphic nature of *C. albicans* (Gulati and Nobile, 2016). It is the most stereotypical fungal pathogen in humans, causing disease ranging from apparent mucosal to lethal disseminated bloodstream infections which make a considerable contribution of more than 40% of mortality rates (Lohse et al., 2018).

The three stages of the development of *C. albicans* comprise adhesion of the yeast cells on the medical devices (early stage), differentiation of the yeast cells to hyphal cells (intermediate stage), and an increase in the matrix which is the maturation phase (Alim et al., 2018). The mature biphasic structure of *C. albicans* is promoted by the adhesive hydrophobic nature of indwelling devices with the proper growing environment. Biomedical devices inserted during transplantation catheter are the favorable ones as they provide nutrition like glucose from the excreted products (Ramage et al., 2006; Alim et al., 2018).

*Candida albicans* colonizes the skin, intestinal mucosa and/or genital mucosa of 30–70% of healthy individuals. Therefore, under normal circumstances, the fungus does not cause any significant disease (Ramage et al., 2014). Furthermore, about 15% of sepsis cases acquired in a hospital due to medical devices occur due to the predominant fungi *Candida* (Lohse et al., 2018). According to some recent reports, 63.5% of patients out of 224 patients suffering from septic shock showed a positive result for *Candida* and were found dead. Thus, it is the leading cause of death in hospital procured diseases (Ramage et al., 2014). In India, just 6-18% of cases of Candidemia are reported. A study exhibits 53 repeated episodes of Candidemia in 48 patients in 1.5 years. Different species made their contribution, like 45% of infections were due to *C. tropicalis*, while 23% of cases by *C. albicans* and the rest 32% by other *Candida* spp. (Kothari and Sagar, 2009). Western countries’ trend on candidemia differs from India as *C. albicans* is related to more than 50% of all candidemia incidences (Kaur et al., 2014).

## Recent Challenge-Drug Resistance

The extensive use of antimicrobials medicaments in a well-connected global human population has increased the cases of antimicrobial resistance (Michael et al., 2014). According to the UN *ad hoc* Interagency Coordinating Group on Antimicrobial Resistance, drug-resistant diseases by 2050 can bring around 10 million deaths per year and cause an economic catastrophe similar to that of the 2008-2009 global financial recession. By 2030, antimicrobial resistance could affect approximately 24 million people plunging them into severe poverty (World Health Organisation [WHO], 2019). According to the Center for disease control and prevention, around 7% of all the blood samples from patients suffering from *Candida* is resistant to fluconazole. However, this resistance to azole is persistent for the last 20 years, but resistance toward echinocandins and other drugs is a huge concern. Thus, this situation will prove to be lethal because of the ever -growing human population.

**FIGURE 1:**
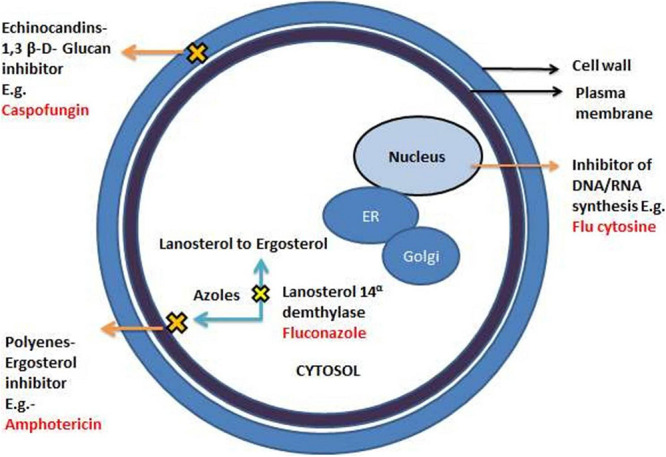
A different mechanism of actions of commonly known antifungal drugs adapted from Spampinato and Leonardi (2013).

Owing to its inappropriate use, antifungal drug resistance has emerged as a major challenge that needs immediate attention. This is further aggravated because of the overuse of antibiotics, which affects the normal microflora of humans, providing a favorable environment for *C. albican* to grow (Ben-Ami et al., 2012). Currently, available drugs are categorized into four major classes which include azoles, polyenes, pyrimidine analogs, and echinocandins. The primary targets of these antifungal drugs are the biosynthetic pathway of ergosterol, the cell wall of fungal cells, or the DNA/RNA of fungi (Borghi et al., 2016; Sagatova et al., 2016; Silva et al., 2017; Figure 1).

Azoles are widely recommended antifungal drugs to treat superficial and invasive fungal infections. The first generation azoles include clotrimazole, bifonazole, econazole, and ketoconazole containing imidazole in their ring system. Fluconazole and itraconazole are some of the renowned examples of second- generation azoles., containing triazole moiety. Voriconazole and posaconazole are the FDA- approved broad -spectrum third generation azole- based antifungal drugs. They exhibit their antifungal effects by inhibiting cytochrome P450-dependent 14α-lanosterol demethylase (Cyp51) encrypted by the ERG11 gene which converts lanosterol to ergosterol (Taff et al., 2013; Silva et al., 2017). The ancient class of fungicidal drugs, polyenes, is used to treat severe infections. It intercalates with the steroids ergosterol, found in the cell membrane, creating pores that obliterate the proton gradient of the cell, destabilizing the cell membrane causing leakage of the ions in the process. Another fungicidal drug, Echinocandins (e.g., Caspofungin), is the newest class of antifungals that acts against *Candida spp.* and is administered intravenously. It interferes with the production of β-1,3- glucans, critical polysaccharides and the major constituent of fungal cell walls (Perfect, 2017; Pristov and Ghannoum, 2019).

Analogs of nucleosides like 5-flucytosine (5-FC) are antimetabolites that imitate nucleotide bases in the course of the synthesis of nucleosides. The 5-Flucytosine acts as a pyrimidine correspondent disturbing the synthesis of fungal RNA, DNA, and protein leading to cell cycle arrest. The 5-FC in itself does not act as an antifungal agent but becomes active when changed into 5-fluorouracil. This conversion of 5-FC into 5-FU is catalyzed by the enzyme cytosine deaminase, residing in fungal cells but not present inside the host cells. Even with a vast diversity of antifungal drugs, the problem of resistance persists against the drug sequestration, making it challenging to fight. The classic inventions, polyenes and azoles, are also ineffective against *C*. *albicans* biofilms, thereby limiting the range of treatment. Thus, the indispensable development of new antifungal therapies with high efficiency against the biofilm mode of growth is required. Biofilms are the major contributors and intensifiers of antifungal resilience. As yet, none of these factors works alone. Instead, this antifungal resilience is a multifactorial phenomenon, which is still unexplored. Various factors of intrinsic resilience which alter normal vegetative cell to more virulent form have been listed briefly. These include an increase in the density of cells within the biofilm, complex association extracellular matrix, the existence of persister cells, gene expression of the antifungal resistance genes, and the proliferation of sterols on the membrane of biofilm cells (Romo et al., 2017; Silva et al., 2017; Su et al., 2018).

## Fungal Biofilms

Fungal biofilms are the complex association of hyphal cells which in turn are associated with both abiotic and animal tissues. They are important virulence factors and correlated with invasive fungal infection (Borghi et al., 2015). They are the sessile microorganisms that, when attached to the abiotic or biotic surfaces, lead to new phenotypic characteristic features (Hawser and Douglas, 1995; Lohse et al., 2018). Implantable medical devices are the favorable sites where *C. albicans* form a complex association forming the biofilms, thus becoming responsible for a proportion of clinical candidiasis (Douglas, 2002).

Furthermore, adherence of the fungal cell to the available biomaterial and its relatedness to bloodstream infections might be due to hematogenous spread. Medical devices provide a perfect niche to yeast cells because of their structure to chemical properties ranging from hydrophobicity to surface roughness. These devices are surrounded by body fluids like urine, blood, saliva, and synovial fluid, which condition them with glycoproteinaceous film (Gristina et al., 1988; Subbiahdoss et al., 2010). This acclimatizing film can accord chemical properties entirely different from its origin. The mature biphasic structure of C. *albicans* is promoted by non-specific factors (cell surface hydrophobicity and electrostatic forces) and specific adhesins on the fungal surface recognizing ligands in the conditioning films, such as serum proteins (fibrinogen and fibronectin) and salivary factors (Chaffin et al., 1998; Demuyser et al., 2014). Also, *C. albicans* cells can co-aggregate to interact with bacterial cells/colonies already vested in these devices (Chaffin et al., 1998; Ramage et al., 2006). Nevertheless, the preliminary focal attachment of the fungal cell to a substratum is accompanied by multiplication and propagation of cells followed by biofilm development (Soll and Daniels, 2016; Silva et al., 2017).

### Pathogenesis of Biofilm

#### Stages of Biofilm Development

**FIGURE 2:**
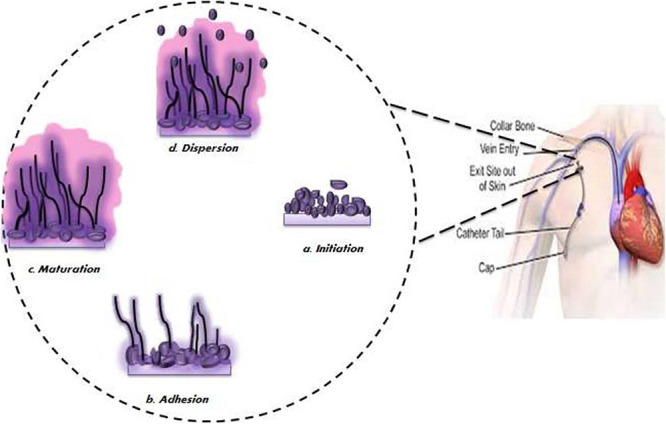
Schematic diagram of different stages of biofilm development.

It has been observed that biofilm development follows sequential steps over a period of 24–48 h (Mathé and Van Dijck, 2013; Taff et al., 2013; Figure 2). Initially, a single yeast cell adheres to the substratum making a foundation for the layer of a yeast cell (*adherence step*) (Finkel and Mitchell, 2011; Mathé and Van Dijck, 2013; Williams et al., 2013). Following this initial phase is the phase of cell proliferation where cells project out and continue to grow into the filamentous structure of hyphal cells through the surface (*initiation step*). The assembly of hyphae marks the beginning of biofilm formation accompanied by the accretion of an extracellular matrix (ECM) on maturation biofilm (*maturation step*). Lastly, non-adhering yeast cells detach themselves from the biofilm into the environment to find a favorable site of attachment (*dispersal step*).

**FIGURE 3:**
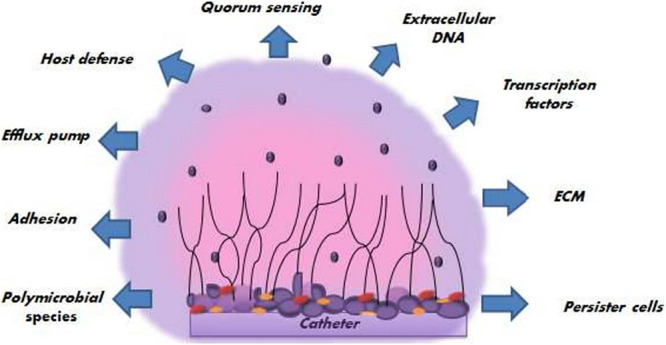
Different factors promoting the pathogenesis of the *C. albicans* biofilm.

Spreading of biofilm-associated yeast cells has tremendous clinical significance as they can start the formation of new biofilms or circulate throughout the host cell and tissues leading to disseminated invasive diseases or candidemia. Various factors promoting the pathogenesis of *C. albicans* biofilm (Figure 3) have been reported, which are discussed in the following section.

#### Extracellular Matrix Formation (ECM)

The extracellular matrix is a critically important feature of biofilms that guards the adherent cells against the host immune system and antifungal agents by forming an extensive structure of the matrix (Borghi et al., 2016). In some of the pioneer works, it was shown that *Candida* species biofilm’s matrices increase when highly dynamic flow environments influence biofilm, and its quantity extensively depends upon strain and the species. Moreover, the chemical composition of *C. albicans* ECM suggests that the extracellular matrix is composed of approximately 55% of a combination of glycoproteins with carbohydrates contributing to only 25% of the total composition. Carbohydrates consist largely of α-mannan and β-1, 6-glucan polysaccharides with β-1, 3-glucans making a very little contribution. ECM also consists of 15% of lipids and only 5% of nucleic acids. β- 1, 3- glucan plays a major role in azole resilience by specific binding. Additionally, the biofilm is two times thicker than planktonic cells. Upon comparison of the chemical composition of planktonic cells with biofilm cells, it was observed that there is a difference in carbohydrate and β 1, 3 glucan composition (Mah and O’Toole, 2001; Nett et al., 2010; Silva et al., 2017; Lohse et al., 2018; Pappas et al., 2018).

#### Extracellular DNA and Genetic Factors

Extracellular DNA (e-DNA) present inside the extracellular matrix is a major contributor to the stability of the *C. albicans* biofilm. The e-DNA is present both in bacterial and fungal biofilms; hence, when these biofilm-forming microorganisms are treated with DNAase enzyme in combination with the respective drugs, they exhibit a decrease in the biofilm matrix (Martins et al., 2014). Various genetic factors like Bcr1 (a transcription factor required for the attachment of fungal cells to the abiotic surfaces), Rlmp, Brg1, Efg1, Ndt80, Rob1, and Tec1, Fsk1p, Smi1p (which works in unification with Fsk1p and Rlmp), Gcr1, Mnn4 are under study. All these factors work together and interact with different genes to regulate and generate biofilms, thus, has given new insight to biofilm formation (Finkel and Mitchell, 2011; Nett et al., 2011; Nobile et al., 2011).

#### Quorum Sensing (QS)

Another mechanism related to extracellular properties of the matrix of the *C. albicans* is quorum sensing, which plays a significant role in the growth of biofilms. It is a density-dependent cell-cell communication mechanism in which autoinducers (signal molecules) are released in response to the increasing density, which enhances or represses the activation of certain genes or factors. This density-dependent mechanism affects different aspects of microorganisms like pathogenesis, morphology, competence, etc. Importantly, it also makes its contribution to biofilm formation. Earlier, QS was thought to be an exclusive feature of a certain bacterial system, but the recent discovery of farnesol, a quorum-sensing molecule inhibiting *C. albicans* biofilms, has publicized QS (Ramage et al., 2002). Genetic regulation of virulent genes in pathogenic microorganisms by QS has shown an indirect connection with the emergence of multi-drug resistant pathogens. Thus, it necessitates the finding of alternative strategies to target QS and restrain the same (Finkel and Mitchell, 2011; Taff et al., 2013).

#### Evasion of Host Immune Responses

The immune system plays a crucial role in the recognition and elimination of the *C. albicans*. The innate immune system, which is the first line of defense, recognizes pathogen-associated molecular patterns of a pathogenic strain of *C. albicans* leading to the activation signaling channels of a host organism resulting in the extermination of the *C. albicans* cells.

According to recent studies, ten diverse surface receptors participate in this recognition pattern. They are Toll-like receptors -TLR4 and TLR2, TLR9, and NLR Family Pyrin Domain Containing 3 (NLRP3) the internal receptors, C-type lectin receptors, Dectin-1and 2, Dendritic Cell-Specific Intercellular adhesion molecule-3-Grabbing Non-integrin (DCSIGN), Mincle, and Mannose-binding lectin. Usually, these receptors recognize and bind to sugar moieties (β-1, 3-glucans, and mannose derivatives) present on the cell surface of *C. albicans*. Due to this binding, the cytokine complement system gets activated, phagocytizing the fungal cells. Internal uptake of these fungal cells by APCs (antigen-presenting cells) expedites the activation of internal receptors; thus, resultant activation of TLR9 or NLRP3 inflammasome occurs. This non-specific immune response (innate response) has a significant role in preventing *C. albicans* infection. Besides, the adaptive immune response also makes its contribution by producing antibodies against some specific extracellular proteins, obstructing the *C. albicans* growth.

On the other hand, there are many strategies employed by *C. albicans* to evade the immune response. Mature biofilms escape immune responses owing to the presence of a top layer of biofilm composed of hyphal cells which mask the β-glucans component of biofilm. Subsequently, these hyphal cells escape the neutrophil killing either by penetrating the epithelial cell layers in the course of invasive growth or by escaping immune response by physical penetrating inside the cell. The variance in the degree of expression of genes in planktonic cells concerning biofilms cells has a connection with a mechanism of immune evasion. There are several proteins that are highly expressed and they also restrict the stimulation of the host complement system. For example, Zinc binding cell surface protein Pra1, a cell surface glycerol-3-phosphate dehydrogenase protein (Gpd2), and all the secretory proteins of aspartyl protease (Sap) family. Another well-known protein that functions as a sensor for cell wall damage with high expression in biofilms is Msb2. This protein secretes and blocks the antimicrobial peptides preventing complement activation.

Neutrophils are the first foot soldiers of the immune system which are altered during biofilm formation. Neutrophils work with different modes of action like phagocytosis, oxidative response, and non-oxidative response against the emerging pathogen. Another known novel mechanism that was discovered in 2004, is the Neutrophil extracellular trap “NETosis.” It is a complex structure that comprises neutrophil chromatin, DNA, and protein. NETosis degranulates neutrophils and releases a lytic enzyme, which is different from necrosis and apoptosis. It has killing properties against planktonic yeast cells; however, it is seemingly ineffective against *C. albicans* biofilms (Branzk and Papayannopoulos, 2013; Johnson et al., 2016, 2017; Tsui et al., 2016). Some studies also suggest that the peripheral mononuclear cells (PBMCs) -due to some unknown factors- thicken the *C. albicans* biofilms rather than phagocytizing them. Furthermore, mature biofilms do not evoke a vigorous oxidative response, a primary mechanism of neutrophil killing microbes (Lohse et al., 2018; Pappas et al., 2018).

#### Polymicrobial Species

Humans provide an immediate small- scale environment to a varied population of microbial species. The human microbiota embraces members from three kingdoms of life: Archaea, Bacteria, and fungi. They usually exist in a symbiotic relationship leading to a complex association of ecosystems. Imbalance in this symbiotic association due to genetic or environmental factors of the host - like changes in pH, shifts in host immunity, and transient viscosity of mucosal layers, and indiscriminate usage of broad-spectrum antimicrobial agents- lead to the disproportionate infection by the overgrowth of specific microbial species over others.

*Candida albicans* is the most frequent fungal pathogen present in commensalism with bacteria. Furthermore, there is incremental progress in the dual-species biofilms formation between common bacterial species and *C. albicans* that usually interact in human beings to cause infection. These kinds of interaction which lead to diseases are studied and concluded based on observational studies, arising mainly in immunocompromised patients. It is difficult to establish a clinical prognosis of polymicrobial interaction from immunocompromised individuals as they are highly vulnerable to other infections. Hence, it is tough to evaluate the degree of polymicrobial interactions and molecular mechanisms from such studies. *In vitro* studies suggest that bacterial species and *Candida spp.* isolated from different parts, like the vagina, oral cavity, etc., interact with each other in different ways. This includes exudation of signaling molecules which influences species behavior toward each other, physical interaction between microbial cells (e.g., hyphal cells provides an attachment site to bacterial cells contained within polymicrobial biofilms), biochemical alterations of the local environment like change in oxygen content and pH. For instance, in the case of the vaginal microbiota, *C. albicans* interacts with Lactobacillus species which produces lactic acid that changes local pH, thus hindering C. *albicans* growth on the mucosal surface of the vagina. Some hyphal cell wall proteins (E.g., Hwp1), transcription regulators (Tec1 and Bcr1, Efg1, Cph1, etc.) and adhesive proteins (E.g., Als1, Als2, and Als3) show a pivotal role in enhancing the expression of virulent genes and hence the resistance in bacterial species such as *Staphylococcus aureus*, *Streptococcus gordonii* and *Staphylococcus epidermidis* (Harriott and Noverr, 2010; Pammi et al., 2013; Chinnici et al., 2019). However, deletion or mutation of these proteins results in a decrease in the number of bacterial cells and weakens their interaction with *C. albicans*. Although biofilms are formed in an oxygen-rich environment, some anaerobic bacteria can easily grow under *C. albicans* biofilms. These biofilms provide bacteria with a positive anaerobic environment to grow. In return, the bacteria augments the formation of *C. albicans* “mini biofilms” which can easily float and grow under the toxic condition (Douglas, 2003; Finkel and Mitchell, 2011; Gulati and Nobile, 2016; Alim et al., 2018).

#### Efflux Pumps

Overexpression of efflux pumps is one of the major contributors to antifungal drug resistance in *C. albicans* as they lead to drug sequestration by pumping out the antifungal drugs given as a treatment. Subsequently, these are primarily concerned with first-generation azoles but ineffective toward Echinocandins resistance. During usual antifungal treatment toward planktonic cells, efflux pumps prevent the intracellular accumulation of antifungal drugs by up-regulating their expression (Borghi et al., 2016; Soll and Daniels, 2016; Tsui et al., 2016). The Cdr1, 2 (ATP binding cassette transporter superfamily), and Mdr1 (Major facilitator transporter superfamily) are the two major classes of efflux pumps that control drug exportation in *C. albicans.* Moreover, initially, after a few hours of up-regulation in biofilms, they persist in being up-regulated all through the biofilm growth even in the absence of the antifungal drug. Rapid upregulation of efflux pumps happens during the primary stage of biofilm formation.

#### Persister Cell and Stress Response

Another major contributing element toward resistance is persister cells, which are the inconsequential subset of yeast cells with minimum metabolic activity assumed to be raised as a phenotypic variant but not a mutant type. They are highly resistant cells within biofilms. Amphotericin B treatment upon *C. albicans* led to the discovery of persister cells. These cells are acknowledged to be persister as they maintain themselves in a dormant stage. Still, in a stressful situation, they reactivate themselves to a state of active metabolism and reinstate as biofilms. Subsequently, drug sequestration by persister cells occurs due to some virulent traits like hyphal growth rather than an expression of efflux pumps and cell membrane structure (Mah and O’Toole, 2001; Tsui et al., 2016; Silva et al., 2017; Lohse et al., 2018).

## Antibiofilm Strategies

The social burden of fungal disease is massive with approximately more than 1.5 billion people affected by the fungal disease worldwide per annum. However, these invasive fungal infections are estimated to be the cause of about 1.5 million deaths per year, making this a noteworthy health problem (Bongomin et al., 2017). While flourishing in its most usual mode of growth, a biofilm of *C. albicans* biofilm displays increased resistance to the available antifungal drugs. These persistent groups of cells are difficult to eradicate and, ever so often, accountable for treatment failures. There is a necessity to develop new strategies to exterminate and treat these emerging *C. albicans* biofilm infections in the medical setting.

Different targets have gained attention aiming to tackle this growing antifungal resistance problem. Treating these biofilms is a big hurdle in medical mycology; thus, there is a vast exigency for the development of new antifungal agents and identification of novel targets.

Considering the efforts undertaken to find solutions to the fungal biofilm infections, the reader may acknowledge the complicated mechanisms that impede the path to reach the panacea. The research was done while managing the patients so far has led to the conclusion that the study of biofilm phenotype is inevitable to restrict fungal biofilms. Besides, the counter effect of fungus on the drug should be examined not just against the planktonic cells but also against the biofilm. Since fully established mature biofilms are much more challenging to treat and eradicate, preemptive practices targeting planktonic cells further inhibiting the early development of biofilms are the need of the hour. Nonetheless, strategies to combat mature biofilms should also be kept in mind. We discuss specific strategies that are under scrutiny, globally, against the problem of drug resistance (Pierce et al., 2015b; Romo et al., 2017).

### Prostaglandins

Molecular studies on biofilms have revealed the important role of lipids on antifungal resistance. The lipid profile of planktonic cells differs from that of biofilms, making lipids a censorious modulator of resistance. This change in the sphingolipids and sterol profile promotes biofilm formation affects cells morphology and physiology altering the adhesion properties in *C. albicans* (Alim et al., 2018; Su et al., 2018). Thus exploiting these prostaglandins adaptation and their properties could be targeted for treatment. The signaling cascade of Arachidonic acid (AA) is pivotal for both biology of humans and fungi morphology and growth. Very nearly each of the following enzymes of the AA cascade can be subjected to the pharmacological investigation: Cyclooxygenase (COX), Cytochrome P 450 (CYP), and Lipoxygenase (LOX) (Liu et al., 2016). Arachidonic acid could be processed into different types of eicosanoids.

Prostaglandins are synthesized by cyclooxygenase enzymes in human cells as well as pathogenic fungi. Prostaglandin E2 is the profusely synthesized prostaglandin that transfers immune responses of humans in the direction which endorses fungal establishment and chronic illness (Erb-Downward and Noverr, 2007; Alim et al., 2018). PGE_2_ inhibits T- helper cells (Type-1) –Th-1 and stimulates the progression of T-helper cells (Type II)-Th-2. These cells are responsible for maintaining the inflammatory responses as Th-1 is accountable for pro-inflammatory responses, while Th-2 for producing anti-inflammatory responses (Berger, 2000). PGE_2_ also down-regulates the production of chemokine and TNFα, thus augmenting the fungal colonization and germ tube formation.

Arachidonic acid, when used along with antifungal drugs, affects the level of prostaglandin E2 in a variety of *Candida* spp. The above-mentioned study suggests that lipid plays a specific role in the structural aspects, signaling cascade, fungal pathogenicity, and metabolic activity of biofilm development in *C. albicans.* However, the defined part of lipid-dependent processes in the development of *C. albicans* biofilm is still not known. Genetic modulation and pharmacological inflection of biosynthetic pathways of lipids are the two stratagems that have been browbeaten in linking lipid alterations with *C. albicans* biofilm morphology- its formation and function in this fungi. Modulating the lipid composition of the cell membrane by comprehensively targeting genes that encode enzymes for lipid biosynthesis regulates endogenic lipid levels (Noverr and Huffnagle, 2004; Erb-Downward and Noverr, 2007; Sherry et al., 2012; Fourie et al., 2016; Liu et al., 2016).

### Modification of the Surface of Biomedical Devices

Biomaterial-associated-infection (BAI) is the major cause of dereliction of biomaterial implants. Microbial contamination of biomaterial devices during implant surgery (peri-operative contamination) or hospitalization (Gristina et al., 1988), causes the onset of BAI. Microorganisms present in BAI are resistant due to the biofilm mode of growth. The extensive use of indwelling catheters in recent medicine, particularly central venous and hemodialysis catheters, has contributed significantly to the increasing incidence of fungal bloodstream infections, in particular, candidiasis (Pierce et al., 2015b).

Traditionally, to decrease the cases of nosocomial infection, which occurs due to central line-associated bloodstream infections (CLABSIs), removal of the devices and providing systemic antimicrobial therapy was performed to eradicate these microorganisms.

These biomedical-assisted devices with a wide range of biomaterials are utilized by a wide range of pathogenic fungi like *C. albicans* to support adhesion, colonization, and subsequent biofilm formation (Ramage et al., 2006; Talsma, 2007). Thus, to preclude these contagious microorganisms on the surface of implanted devices with biofilms, there is an inquisitiveness in the development and improvement of unconventional biomaterials which are unfavorable to microbial (both fungal and bacterial) adhesion and colonization. Another modification strategy from some studies suggests that modifying the surface chemistry of the biomaterials could be an approach to prevent or reduce biofilm formation. This could be achieved by adding surface-modifying end groups (SMEs) or by altering the chemical composition of substrates. For example, SME Polyetherurethane when added to Elasthane 80A, a biomaterial, decreased the *C. albicans* ability to form biofilm significantly (Chandra et al., 2005).

### Antibiotic Lock Therapy (ALT)

Antibiotic lock therapy is, in general, a combination of an antibacterial solution that has minimum inhibitory concentrations (MIC) 100-1000 times greater than the antibiotic used for planktonic cells in combination with the anticoagulant instilled into the lumen of a catheter. The solution is allowed to dwell or is “locked” while the Central Venous Catheter (CVC) is not used to prevent colonization or sterilize a previously infected catheter (Bookstaver et al., 2013; Justo and Bookstaver, 2014; Norris et al., 2017). The study suggests that only 10% of the patients subjected to ALT needed conventional replacement therapy compared to 33% of the patients treated with systemic antimicrobial drugs (O’Horo et al., 2011). Ethanol Lock solution at more than 40% dwelled onto catheter with *C. albicans* biofilm cleansed the catheter within 30 minutes (Öncü, 2014). Interestingly, an *in vitro* study of 40% ethanol lock solution in combination with 60IU heparin showed a significant decrease in ≥90% of *C. albicans* biofilm metabolism (Alonso et al., 2018).

### Small Molecular Inhibitors and Natural Products

Microbiological resistance depends on various fungal factors that have been established due to genetic alteration in the fungi. Clinical resistance is due to host- or drug-related factors. All these factors may cause fungal resistance individually or in tandem. In addition to standardized susceptibility testing and appropriate drug dosing, one way to avoid resistance is the use of combinational antifungal therapy. Combination therapy also offers advantages in increased synergistic action with enhanced spectrum activity. Newer insights into mechanisms of drug resistance will help in the development of appropriate antifungal therapy.

For dealing with this problem of resistance, there are two ways: (i) finding of the novel anti-biofilm molecules and (ii) repurposing of the known drug to increase the activity of antifungal agents by combinational therapy. Due to the scarcity of known molecular scaffolds that inhibit/disperse fungal biofilms, high throughput screening (HTS) has been employed in an attempt to discover leads for new anti-biofilm modulators. Intending to identify novel small molecules that possessed anti-biofilm activity, screening of an extensive chemical library of compounds took a new start. It was observed that clinical isolates of *C. albicans* have varied abilities to respond to different growth media to form hyphae and also biofilms, and this variability is not associated with specific host conditions or characteristics. A high-content screen has identified many new structural series of antifungal compounds with the mode of action, namely inhibition of biofilm formation and filamentation. Many compounds among them represent a promising candidate for the development of novel anti-virulence approaches against *C. albicans* infections, aimed at minimizing the development of resistance.

#### Different Targets of Small Molecule Inhibitors

Small molecular inhibitors have been identified specifically for various targets along with their MIC/IC50 values which are summarized in the tables mentioned below.

##### Inhibitors of budded to hyphal transition

The pathogenic yeast *C. albicans* can exist in multiple morphological states, including budded, pseudohyphal, and true hyphal forms. The ability to change between the budded and hyphal forms, termed as the budded-to-hyphal-form transition, is important for virulence and is regulated by multiple environmental and cellular signals (Toenjes et al., 2005).

Filastatin **1,** inhibits yeast to hyphal transition, impedes the adhesion of fungal cells to different biomaterials by inhibiting the hyphal specific *HWP1* promoter. Interestingly, in the *in vivo* studies, filastatin in the presence of fluconazole protected *C. elegans* against *C. albicans* infection. Moreover, it also inhibited biofilm formation in a mouse model of vulvovaginal candidal infection (Fazly et al., 2013). Similarly, Johnson and the group identified several small molecular inhibitors. This includes Buhytrin A **2**, CGP-37157 **3** (affecting the calcium metabolism of bud to hyphal transition), and ETYA **4** (inhibiting multiple signaling pathways) and Clozapine **5** (FDA approved antipsychotic drug inhibiting Efg1 pathway at Gpr1 G-protein-coupled receptor level). These molecules restrict the bud to hyphal growth in *C. albicans* and affect multiple signaling pathways that are intricate in the filamentation process which regulates the biofilm formation (Midkiff et al., 2011; Grald et al., 2012; Pierce et al., 2015b). By screening a library of 50,240 small molecules of inhibitors for yeast-to-hypha transition, Sm21 **6** was identified as a novel small antifungal molecule that showed its efficacy in both *in vivo* oral candidiasis mouse model and *in vitro* system. The study also revealed that compound **6** when given to *C. albicans* strain led to reactive oxygen species (ROS) accumulation and mitochondrial dysfunction (Wong et al., 2014; Truong et al., 2018). In another study, Chrysazin **7** (1, 8-dihydroxyanthraquinone) and Alizarin **8** (1, 2-dihydroxyanthraquinone) an anthraquinone derivative, effectively inhibits biofilm formation in *C. albicans.* Their anti-biofilm activity was mainly due to a hydroxyl group (-OH) at C1 position. Unlike other commercially available drugs, alizarin was found to be a non-toxic compound. It downregulates the expression of various hyphal-specific and biofilm related genes like *ALS3, ECE1, ECE2*, and *RBT1*. Additionally, Chrysazin and Alizarin at 2 μg/ml adequately inhibited yeast to hyphal formation and increased the survivability of *C. albicans* infected *C. elegans*, thus, proving to be a strong candidate for future investigation as an antifungal agent (Manoharan et al., 2017b).

While performing high throughput screening in a chemical library (NOVACore^TM^), about 20,000 small molecules were identified as a novel series of a Diazaspiro-decane and its structural analogs (9a-d). These molecules inhibit progressions of the virulence-associated with *C. albicans*, especially biofilm formation and filamentation, without affecting the overall growth or prompting the resistance in *C. albicans.* These compounds displayed a potent inhibitory activity when tested against murine models of oral candidiasis (Pierce et al., 2015a). In addition to all these, when 678 small molecules were screened from the chemical library by the group scientist against the invasive hyphal growth of the opportunistic human yeast *C. albicans*, it led to the discovery of two halogenated compounds (N1-(3,5-dichlorophenyl)-5-chloro-2-hydroxybenzamide) 10 and Niclosamide 11 which is the analog of salicylanilide, an FDA-approved anthelmintic in humans, both exhibiting an anti-filamentation and anti-biofilm activities against *C. albicans* (Garcia et al., 2018).

Another compound N-[3-(allyloxy)-phenyl]-4-methoxyben zamide **12** was recognized as the lead one showing inhibition against C. *albicans* filamentation and was found effective in both *in vitro* and *in vivo* study of *C. albicans* infected murine model. The whole transcriptomic analysis revealed a total of 618 genes that were up-regulated and 702 were down-regulated. It was observed that most of the down-regulated genes such as SAP5, ECE1, and ALS3 already well-characterized were associated with filamentation and virulency. In addition, some genes affect metal chelation and utilization (Romo et al., 2019).

Homology studies using *Candida* Genome Database showed that *Candida* accounts for type III 5-α-reductase, Dfg10p, and shares a 27% sequence identity and 41% similarity to the human type III 5-α-reductase, which were identified as a target for benign prostatic hyperplasia. Finasteride **13** was recognized as one of the potent inhibitors against type III 5-α-reductase. Studying its activity showed some promising results at dose ≥16 mg/liter. Finasteride alone was highly efficient in preventing filamentation of *C. albicans* and demonstrated synergy with FLC against *in vitro* urinary biofilm (Chavez-Dozal et al., 2014).

LaFleur group, while filtering through 120,000 small compounds from the NIH Molecular Libraries Small Molecule Repository, identified 1,3-benzothiazole **14, 14(a-c)** and its scaffolds as an anti-filamentation drug which acts as an potentiate a known antifungal drug Clotrimazole by increasing its activity to >100 fold against *C. albican* biofilm isolates (LaFleur et al., 2011).

##### Enzymes

Potent inhibitors of essential microbial enzymes are significant growth inhibitors of *C. albicans*, a pathogenic fungi. The enzyme aspartate semialdehyde dehydrogenase (ASADH) is critical for the functioning of the biosynthetic pathway of aspartate in microbes and plants, an important step for the biosynthesis of other essential amino acids. Because the aspartate pathway is absent in humans, ASADH can be a promising new target for antifungal research. Deleting the ASD gene encoding for ASADH significantly decreases the survival of *C*. *albicans*, establishing this enzyme as essential for this organism (Dahal and Viola, 2018; Dahal et al., 2020).

The 1,4-Napthoquinone and its derivatives **15, 15 (c-e)** did possess activity against clinical isolates of *C. albicans* derived from gynecological patients by inhibiting phenotypic changes in *C. albicans*. Additionally, some other studies also establish that dichloro-derivatives of 1,4 naphthoquinone i.e., 2-chloro 1,4-Naphthoquinone **15a** and 2,3-dichloro naphthoquinone **15b** are the inhibitors of ASADH (aspartate dehydrogenase) (Janeczko et al., 2018).

Secreted Aspartic Protease-2 enzymes (SAP2) of the *C. albicans* are one of the known classes of the virulent factor for localized and systemic infection. In *C. albicans*, ten distinct SAP genes (SAP1-10) were expressed *in vitro* and *in vivo*. The SAPs are essential for the fungal nutrition process and contribute to the fungal pathogenicity due to their critical participation in several stages of the infective process, including adhesion, invasion, and tissue damage and so on. Pepstatin and peptidomimetic are the known peptide inhibitors, but it is challenging to clinically synthesize them due to pharmacokinetic characteristics. So from the studies, compound **16,** one of the derivatives of pepstatin, came out to be a potent inhibitor against 3 of the SAP2 present in fungi. Moreover, compound **16** and fluconazole when administered to FLC resistant mouse model infected with *C. albicans* showed synergistic effect by increasing the survival rate of the mouse by 50% (Cadicamo et al., 2013).

Targeting proteinases and phospholipases, which are vital to fungal invasion of host tissues and immunosuppression, Aneja et al. (2016) developed an s triazole series–amino acid hybrids. It was found that compounds **17** and **18** significantly reduce the secretion of proteinases and phospholipases in *Candida* spp. which are vital to fungal invasion of host tissues and immunosuppression. The study showed that on treatment with compound **17**, proteinase secretion was decreased by 29, 22 and 23.5% in standard, FLC-sensitive and resistant strains of *C. albicans*, respectively, while at the same concentration, compound 18 decreased the proteinase secretion by 30, 33 and 17% against the respective strains. At similar concentrations in the same strains, compound **17** caused 40, 38, and 38% inhibition in phopholipase secretion whereas compound **18** decreased the secretions by 36, 27 and 38% respectively.

##### Efflux pump

One of the structural derivatives of cyclobutene-dione [3-(phenylamino)-4-{[1,3,3-trimethyl-2,3-dihydro-1H-indol-2-ylidene]methyl}-cyclobut-3-ene-1,2-dione] **19** was chemically synthesized and was identified as the puissant inhibitor of the efflux pumps residing in plasma membrane - Major Facilitator Superfamily (MFS) and ATP-binding cassette (ABC) transporters accountable for efflux pump-mediated drug resistance in the fungal pathogen *C. albicans* (Keniya et al., 2015).

#### Quorum Sensing Molecules

As already mentioned, quorum sensing plays an important role in mediating the formation of biofilm of *C. albicans*. One of the known quorum-sensing molecules released from *Candida* itself, “Farnesol” **20,** is known to inhibit filamentation in *C. albicans*, which is a known virulence factor enhancing biofilm formation. From the previous studies it is known that the upregulation of the sterol biosynthetic pathway gene *ERG* and the efflux pump genes *CDR* and *MDR* are one of the major contributors to the azole tolerance in *C. albicans*. Therefore, through northern blot studies, it was examined that Farnesol down-regulates the expression of partial gene expression in ergosterol biosynthesis, Ras1-cAMP-Efg1 signaling cascade, *HWP1* promoter, which encodes for hyphal –specific cell wall protein (Ramage et al., 2002; Yu et al., 2012; Dižová and Bujdáková, 2017). Further, it was observed that compound **20** is in synergism with already known antifungal drugs augmenting the accumulation of ROS, leading to early apoptosis in fungal cells (Sharma and Prasad, 2011). In a recent study, a bacterial quorum sensing quencher S8- Thiazolidinedione-8 **21** has been identified as an effective small molecular inhibitor which significantly decreases biofilm formation by *C. albicans.* Additionally, it shows, anti-filamentation, as well as anti-adhesion activity at four to eight fold decreased MIC concentrations. Nevertheless, it down-regulates *HWP1, ALS3, EAP1*, *HST7* and *CPH* transcription factors that play a key role in biofilm formation (Feldman et al., 2014). Another newly discovered Thiazolidinedione molecule- N-(oxazolylmethyl)-thiazolidinedione **22** scaffolds has been identified as a novel compound that inhibits *C. albicans* Als surface protein (Marc et al., 2018; Table 1).

**TABLE 1:** Chemical structures and MIC/IC_50_ values of small molecule inhibitors (1–22).

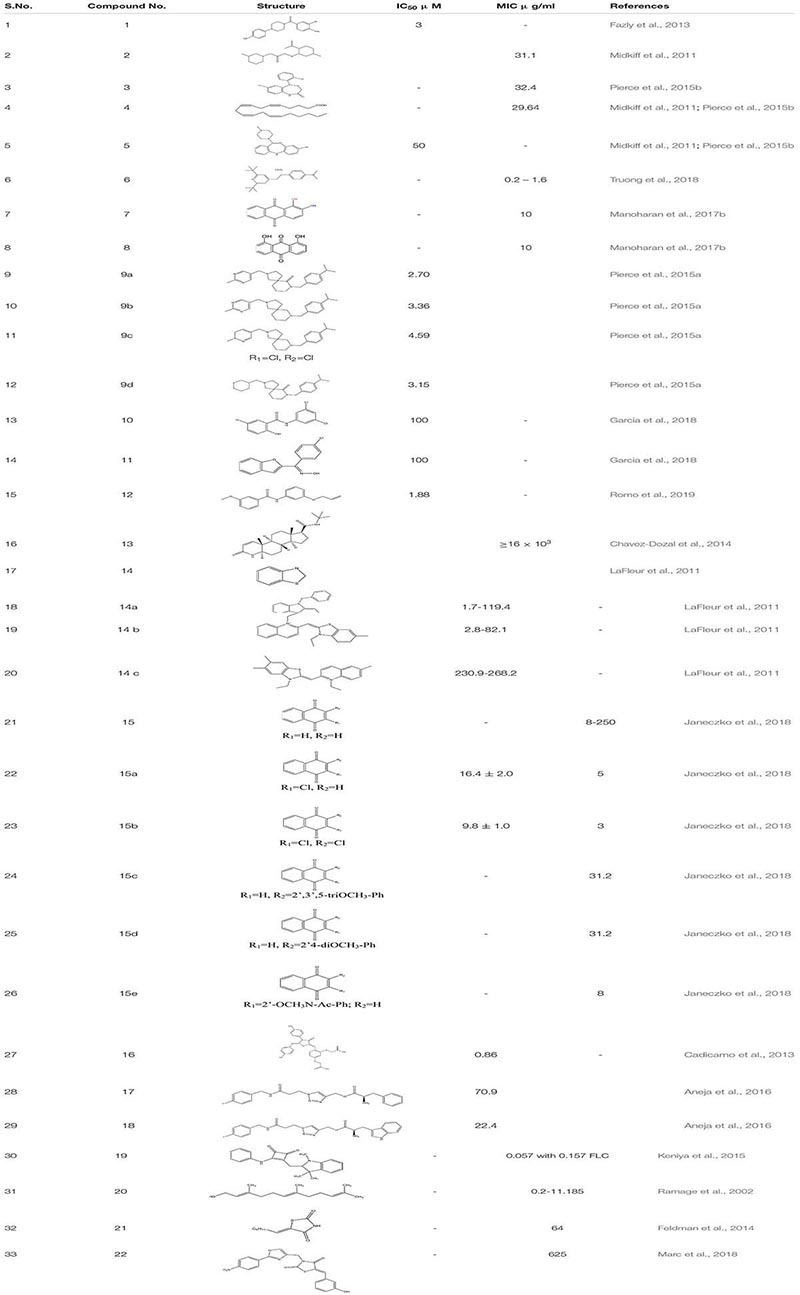

#### High Throughput Screening of the Libraries

Around 400 heterogeneous drugs like molecules assembled by Medicines for Malaria Venture [MMV], Switzerland was screened with the motto to hasten the identification of medicinal molecules *C. albicans* biofilm. Three confirmed hits were obtained- MMV688768, MMV687807, and MMV687273, which exhibited activity against pre- biofilms. However, by additional exploration, it was observed that compound MMV688768- 2-methyl-3-[(4-methylpiperazin-1-yl)-thiophen-2-ylmethyl]-1H- indole **23** an anti-*Schistosoma* drugs are the best showing anti-biofilm activity against *C. albicans*, with concentrations as low as 3.12 μM (Vila and Lopez-Ribot, 2017).

Ebselen **24** an organoselenium compound, was screened out from the off-patent drugs using the Prestwick Chemical Library, a repurposing library of about 1,280 small molecules. Out of nine molecules, Ebselen illustrated 100% inhibition against multi-drug resistant species, *Candida auris* at concentration ≤ 2.5 μM (Wall et al., 2018). Furthermore, it elucidates its antifungal activity by accumulating reactive oxygen species (ROS) inside the fungal cells. Further, *in vivo* study of *C. elegan* model infected with *C. albicans* showed complete eradication of fungal load when treated with 8 μg/ml of ebselen (Thangamani et al., 2017). Additional screening of New Prestwick Chemical Library comprised of FDA-approved 1,200 off-patent drugs. Alexidine dihydrochloride **25** (AXD) and Thimerosal **26** showed more than 80% of inhibition mature biofilms. Subsequently, AXD showed antibiofilm potency *C. albicans* mouse model disseminated with fungal infection (Mamouei et al., 2018; Table 2).

**TABLE 2:** Chemical structures and MIC/IC_50_ values of small molecule inhibitors (23–26).

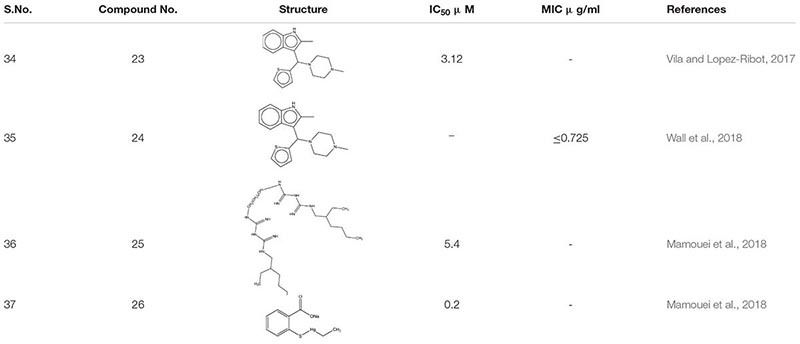

#### Phenotypic Screening

Phenotypic screening refers to the nascent methodology for the biological screening of the chemical entities to assess their therapeutic effects. It is not a target- based strategy but involves detailed cell-based studies (Aulner et al., 2019). The phenotypic screening was done to identify different small molecules which depict antibiofilm and antifilamentation activity against *C. albicans.* This study leads to the identification of about 2293 compounds from the chemical library of the National Cancer Institute which were categorized into three sets- (i) NCI Natural set, Out of all the compounds present in this set six hits were confirmed against *C. albicans* biofilm formation. These include -Trichoderonin **27**; Nanaomycin **28**; Rapamycin **29**; Anisomycin **30**; Valinomycin **31** and Bacitracin **32**. Three of these molecules (Trichoderonin, Nanaomycin, Rapamycin) showed inhibition of both filamentation and biofilm formation while the rest three showed inhibition against biofilm formation.

Furthermore in the next (ii)-NCI-Structural diversity set, in total, there were 12 hits out of which eight were identified as biofilm inhibitor. These compounds were-Phenanthroline Hydrochloride **33**; 2-isoquinolin-2-ium-2-yl-1-phenanthren-3- ylethanone, iodide **34**;Metanilamide (3-aminobenzenesul fonamide) **35**; Mercury, (4-amino phenyl)(6-thioguano sinato- N7,S6)- **36**; 2-[7-[3-(carboxymethyl)-5,10-dihydroxy-1-methyl- 6,9-dioxo-3,4-dihydro-1H-benzo[g] isochromen-7-yl]-5,10-dihy droxy-1-methyl-6,9-dioxo-3,4-dihydro-1H-benzo [g]isochro men-3-yl]acetic acid **37** are only biofilm inhibitors while Mercury,(2-aminio-1,9-dihydro-6H-purine-6-thionato-N7,S6) hexyl-,2-benzo[a]phenothiazin-12-yl-N,N-diethylethanamine **38**; 17-[1-[2(dimethylamino)ethylamino]ethyl]-13-methyl-6,7,8, 9,11,12,14,15,16,17 decahydrocyclopenta[a]phenanthren-3-ol **39**. Three of these compounds inhibited both filamentation and biofilm formation. From all these compounds related to 17- aminoestradiols, a Mercury containing organometallic was the most potent compound with IC_50_ value in the range of nanomolar concentration.

Next and last was (iii)-NCI-Challenge Set. In this, there were total 11 hits, of which 10 showed inhibition against *C. albicans* biofilm formation whereas only one compound inhibited filamentation transformation. Ten hits which were identified from these compounds displayed common inhibition against both biofilm and filament formation. These include Biofilm Inhibitor- Trichopolyn-B**40**, Vengicide (Unamycin B, Toyocamycin)**41**, 4Z-4-[[4-(dimethylamino)phenyl]methyli dene]-1-methyl-2-phenylpyrazolo[1,5-a]indol-1-ium-6-ol;trifluo romethanesulfonate**42**, Anisomycin**43**, Azetidinecarbo thioic acid, [1-(2-pyridinyl) ethylidene] hydrazide**4**. Additionally, compounds with both antifilamentation and antibiofilm activity are.- 6-Hydroxy-3-((methanesulfonyloxy) Methyl)-1- ((5,6,7-tri methoxyindol-2-yl) carbonyl)indoline**45**, Hydrazineca rbothioamide, N, N-dipropyl-2-(2-pyridinemethylene)-,(N,N,S) copper(II)chloridecomplex(SP-4-3)3;3-Azabicyclo[3.22]nonane-3-carboselenoicacid,[1-(2pyridinyl)ethyidene] hydrazide**46**, 2- hydroxyethyl-[(2R)-2-hydroxyheptadecyl]-dimethylazanium iodide **47**, 1H-Azepine-1-carbothioic acid, hexa hydro-, [1-(2-pyridinyl) ethylidene]hydrazide**48** (Pierce et al., 2014; Table 3).

**TABLE 3:** Chemical structures and MIC/IC_50_ values of small molecule inhibitors (27–48).

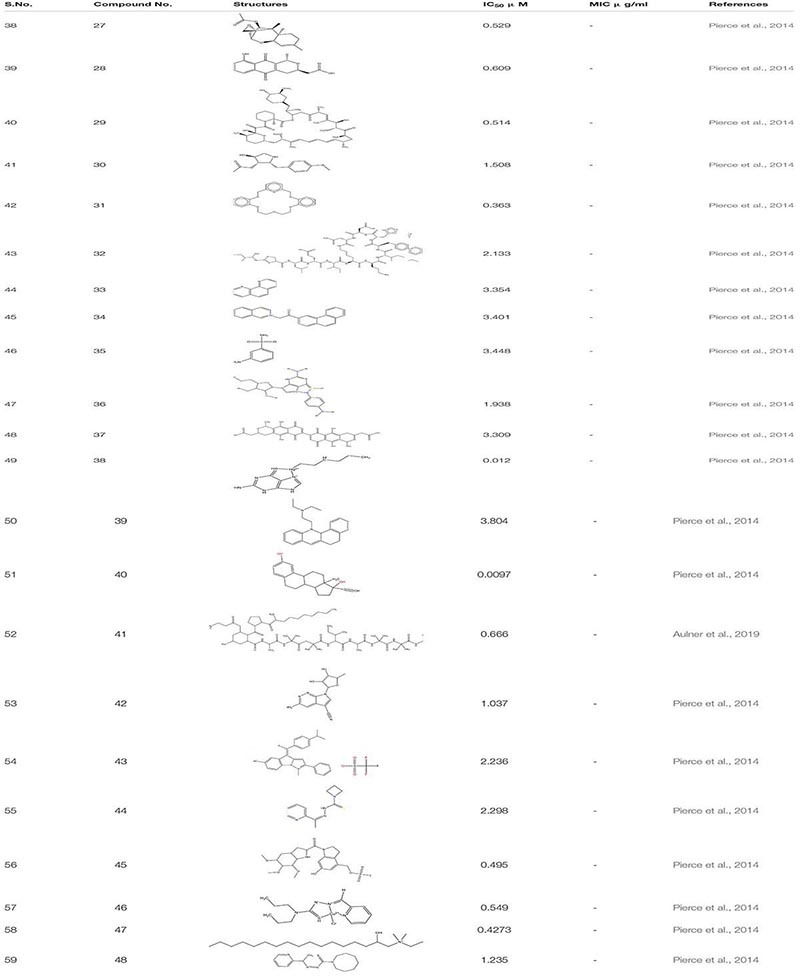

#### Repurposing FDA Approved Drug Showing Synergistic Effect

Haloperidol **49** and its derivative (Benzocyclane derivative B10 **49a)** (Ji et al., 2020) and Bromoperidol **50** and its derivative are some of the known antipsychotic drugs that were repurposed and were utilized as anti-biofilm molecules. Haloperidol’s derivative exhibited inhibition of yeast to hyphal transition in resistant strain of *C. albicans* in synergism with FLC while it also down-regulated the expression of ERG11, and MDR1 genes are leading to cell membrane damage. Moreover, compound **49a** showed significant decrease in the CFU/g from 7.63 to 7.29 in murine model of invasive candidiasis. Similarly, Bromoperidol and its derivative showed a synergistic effect in combination with different azoles. The mechanism of action is unknown but it does decrease the amount of azole administered at the time of treatment (Holbrook et al., 2017).

While looking for different antibiofilm drug groups, scientists looked for the Hsp 90 and Calcineruin inhibitors as it chemo sensitizes the fungal strains toward azole- as the first line of antifungal drugs. Piperazinyl quinolone **51** screened from MLPCN library had no antifungal activity alone, but, in combination with Azoles, it selectively inversed the fluconazole resistance in the clinical isolates of *C. albicans* (Youngsaye et al., 2011). Similarly, FK506 **52** and Cyclosporine A **53**, the other potential calcineruin inhibitors also illustrated the increase in the susceptibility of *C. albicans* toward antifungal drugs (Uppuluri et al., 2008; Cordeiro et al., 2014; Jia et al., 2016). Ribavirin **54,** already known antiviral drug, was screened through Prestwick Chemical Library against FLC-resistant strains. It illustrated a fungistatic effect against MDR species of *C. albicans* and acted synergistically with MIC < 24.4 μg/mL (Yousfi et al., 2019).

While going through 1,600 compounds present in the drug-repositioning library, 8 hits were obtained. These are Hexachlorophene **55** (anti- topical anti-infective drug) (Siles et al., 2013), Pyrvinium pamoate **56** (an anti-helminthic drug), Artesunate **57** (antimalarial drug), Broxyquinoline **58** (antiseptic drug), Dihydroartemisinin **59** (antimalarial drug), Gentian violet **60** (antibacterial and antihelminthic drug), Bithionate disodium **61** (antiseptic), and Nitroxoline (Antibacterial). Out of these 8 hits, the first seven hits showed inhibition of mature *C. albicans* biofilm in combination with miconazole at sub-inhibitory concentrations. Further, studies were performed on the best-acknowledged potentiators, Hexachlorophene, pyrvinium pamoate, and artesunate. Mechanistic synergy was most pronounced for artesunate, an artemisinin homolog, which prompted the study of different structural homologs of Artemisinia. Thus, it was observed that biofilm inhibition in combination with Micanazole was not specific only to artesunate but also applicable to dihydroartemisinin and other artemisinin derivatives, indicating artemisinin as a potential antifungal molecule that can be further investigated to establish its overall potentials in the human health care system (De Cremer et al., 2015).

In another study, the library comprehending 1600 off-patent drugs was sifted for the compound that enhances *in vitro* activity of amphotericin B when used in combination against *C. albicans* biofilms. Out of 1600 compounds, the team found 50 hits, and from these 50 hits, only seven of them illustrated BIC_50_ < 100 μM for biofilm inhibition. Those compounds are as follow: Prochlorperazine edisylate 5.2 μM **(62)**, Danthron 12 μM **(63)**, Chlorprothixene hydrochloride 17 μM **(64),** Toremifene citrate 19.5 μM **(65)**, Clorgiline hydrochloride 24 μM **(66)**; Perhexiline maleate 39 μM **(67)** Dicyclomine hydrochloride 60 μM**(68)**. Subsequently, all of these compounds showed a synergistic effect with caspofungin. Additionally, while studying tormifene citrate **(65)** for its synergistic effect on *C. elegans* infection model, it showed strong antifungal potency (Delattin et al., 2014). Diclofenac **69** a non -steroidal anti-inflammatory drug, increased the susceptibility of caspofungin toward the *in vitro C. albicans* biofilm. Furthermore, catheter retrieved from the animal model-rat when treated with combination therapy of caspofungin and diclofenac showed a significant reduction in the number of biofilm cells (Bink et al., 2012). Further, an off-patent antipsychotic drug Aripiprazole **70** was identified as a potent inhibitor of *C. albicans* which inhibited the early formation of pseudohyphal cells and mimicked standard azoles at higher concentrations showing different mechanisms of action (Rajasekharan et al., 2019; Table 4).

**TABLE 4:** Chemical structures and MIC/IC_50_ values of small molecule inhibitors (49–70).

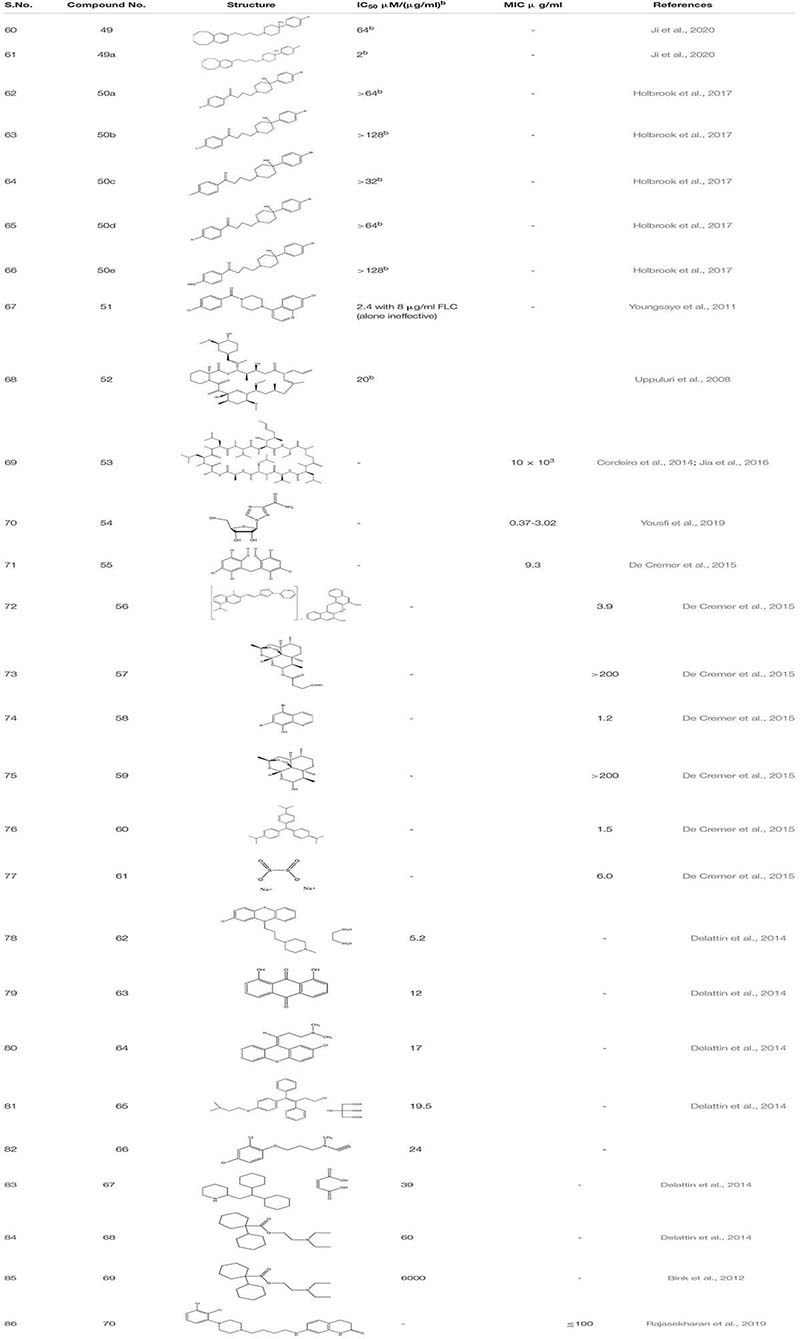

*^*b*^means that the unit of IC_50_ value is μg/ml.*

### Natural Products

Arising fungal resistance ascribed to mutational changes has diversified the complexity fueling the thought processes along this line. To overcome this issue, we need to find some substantial solutions. For the past few decades, natural products have emerged as an essential source of antibacterial, antimalarial, and chemotherapeutic agents. Presently, around 60% of used drugs for cancer treatment are procured from natural products. Furthermore, the modification of natural products is one of the most common and productive methodologies to obtain novel therapeutic agents using medicinal chemistry (Zaki et al., 2019). Thus, targeting fungal biofilms by either natural derivatives or synthetic analogs could be a novel approach. Natural product screening has proved to be one of the promising strategies. Although anti-biofilm agents themselves might not kill the bacteria, they can make them more susceptible to conventional antibiotics and the action of the host immune system. The search for biofilm inhibitors has led to identifying a significant number of compounds of potential therapeutic use as biofilm inhibitors. A literature survey reveals that some of the most active anti-biofilm compounds discovered to date have been based upon the molecular scaffolds of natural products isolated from marine natural products and plant products (Fazly et al., 2013). As compiled by the World Health Organization, more than 21,000 plant species containing a vast array of biologically active compounds have been used worldwide in herbal medicines (Midkiff et al., 2011). Naturally occurring alcohols magnolol **71** and its isomer honokiol **72**, carvacrol **73**, thymol **74,** and geraniol **75** have been identified as a potent inhibitor of both bacterial and *C. albicans* biofilms (de Castro et al., 2015; Magi et al., 2015; Sun et al., 2015; Sharma et al., 2016; Behbehani et al., 2017). Furthermore, when *C. elegan* was treated with 16 μg/ml of magnolol and honokiol, there was a decrease in the number of colony forming units (CFU) of *C. albicans* in the nematode which enhances its survival. Additionally, antibiofilm activity of these compounds **69** and **70** were related to Ras1-cAMP-Efg1 pathway of *C. albicans* (Sun et al., 2015). A Recent study shows that compound **70** and **71** are antifungal and induce ROS mediated programmed cell death in *C. albicans* (Sun et al., 2017; Niu et al., 2020). Saponins are one of the known natural products that bind to the ergosterol rather than cholesterol of *C. albicans.* This increases the susceptibility to photodynamic inactivation due to an increase in the permeability of photosensitizers by sapnonin. In one study, a total of 12 different saponins were identified as potential antifungal agents, but out of those 12, only two were selected. One was **76,** which belongs to the aginoside family of saponins and the secone one **77** to the barrgeniol family of the saponins. Both of these compounds were potent antifungal agents with MIC 16 and 32 μg/ml, respectively. Furthermore, to study toxicity and efficacy of the compound, *C. elegans* and *C. albicans* models were used. In an *in vivo* study, compound **76** and **77** showed no sign of toxicity and conferred high percentage of nematode survival from upto 73% to 80%, respectively (Coleman et al., 2010).

Occasionally, other known naturally occurring compounds are found to be useful. The Carvone **78** and perillaledhyde **79** (McGeady et al., 2002; Tian et al., 2017; Moro et al., 2018) at very low concentration inhibit the filamentous formation of *C. albicans*. Furthermore, it was observed that at MIC of 4 μl/mL compound **77** increased the level of ROS, which activated the programmed cell death in *C. albicans*. Additionally, it equalizes the level of E-cadherins the epithelial barriers preventing which decreases in number due to invasion of pathogenic fungi (Tian et al., 2017; Qu et al., 2019). The Riccardin D**80, 80a** - macrocyclic bisbibenzyl isolated from *Dumortiera hirsute* chinese liverwort, exhibited an inhibitory effect on the biofilm formation of *C. albicans* by downregulating HWP1, ASL3, and EFG1 genes thus, inhibiting hyphal formation. Additionally, one of the semi-synthetic derivative of Riccardin D with a bromine atom attached to the arene ring showed better antifungal activity with MIC-2 μg/ml in comparison to Riccardin with MIC-16 μg/ml (Li et al., 2012; Sun et al., 2016). Another known compound is Emodin **81** (1, 3, 8−trihydroxy−6−methyl−anthraquinone) which is a natural secondary plant product, originally isolated from the rhizomes of *Rheum palmatum*. This compound suppressed the growth of the cells of reference and clinical *C. albicans* strains with minimal inhibitory and minimal fungicidal concentration values between 12.5 and 200 μg/mL, respectively, showing anti-virulent potential. Research showed that emodin added to *C. albicans* culture inhibited the phosphorylation of many cellular proteins, presumably, owing to the inhibition of protein kinase CK2. Notably, the enzyme isolated from the *C. albicans* cells was found to be susceptible to emodin with IC50 of 2.8 μg/mL, revealing that emodin was able to occupy the ATP−binding pocket of CK2 (Janeczko et al., 2017). In another finding, *M. sylvestris* root was also shown to inhibit *C. albicans* biofilm formation (Alizadeh et al., 2017). Studies on quorum sensing inhibition and the quest for QS inhibitors have shown plants to produce anti-QS substances. Several anti-QS methods have been used, including natural products from plant-based secondary metabolites (Berde et al., 2019). Carbohydrate derived fulvic acid (CHD-FA) **82** from pure form of fulvic acid is obtained from humic substances, also exhibits anti-biofilm activity against *C. albicans* (Sherry et al., 2012; Borghi et al., 2015). Plant alkaloid berberine **83** showed 91% biofilm inhibition against *C. albicans* in combination with Miconazole. Furthermore, it reduces the metabolic activity of early- stage biofilm formation in *C. albicans.* Individually, Berberine inactivates fungal biofilm at MIC range 0.98-31.25mg/mL (Wei et al., 2011). Resveratrol (3, 4, 5 -trihydroxystilbene) **84** is a phytoalexin, a known antimicrobial agent present in a wide range of dietary sources, including peanuts, plums, grapes and in red wines. A semisynthetic compound -EB487 **84a** was synthesized from resveratrol, which showed an antibiofilm and anti-preformed biofilm activity against *C. albicans* strain. Subsequently, the study revealed that resveratrol regulates its antifungal activity from early to late apoptosis stage. Furthermore, it increases the ROS concentration in fungal cells of *C. albicans* and modulates the activation of metacaspase release in response to mitochondrial dysfunction (Lee and Lee, 2015; Juin et al., 2019; Table 5).

**TABLE 5:** Chemical structures and MIC/IC_50_ values of small molecule inhibitors (71–84).

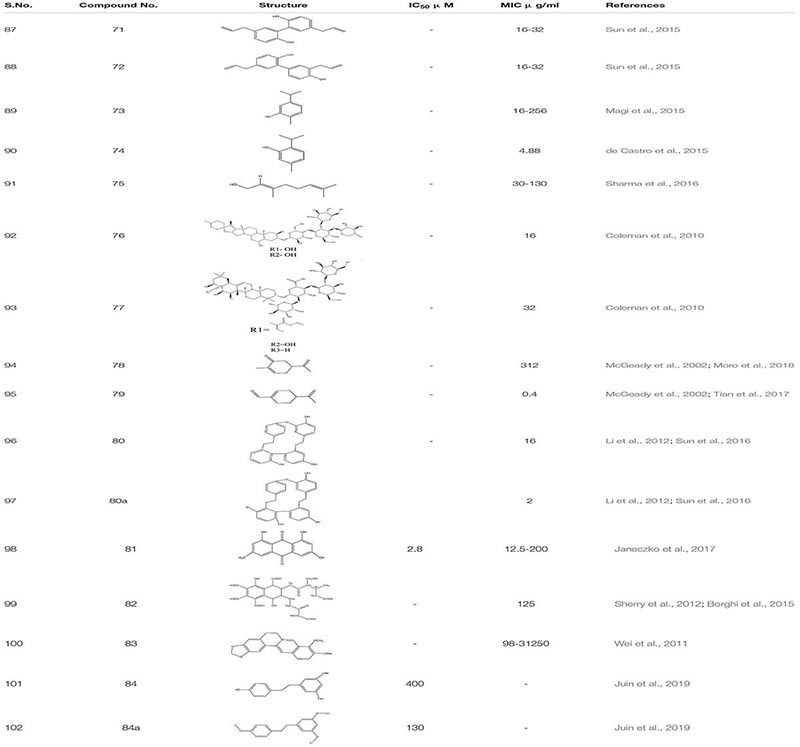

#### Natural Extracts and Essential Oils

Ethanol extract of *Tovomita krukovii* led to the identification of few new xanthones like 1,3,5-trihydroxy-8-isoprenylxanthone **85a**, 1,5,7-trihydroxy-8-isoprenylxanthone **85b**, betulinic acid **86,** and 3,4-dihydroxybenzoic acid **87** targeting secreted aspartic protease SAP2 of *C. albicans* with IC_50_ values 15 μg/ml, 25 μg/ml, 40 μg/ml, and 6.5 μg/ml, respectively (Zhang et al., 2002). Crude hydro alcoholic extract prepared through peel extract of *Punica grantum* presented strong inhibitory activity against *C. albicans* at MIC 3.9 μg/mL and led to the two-fold decrease in the concentration of FLC when used in combination (Endo et al., 2010). In certain studies, essential oils from plants and their compounds have been scrutinized as antifungal agents. The essential oil of *Coriandrum sativum* delineates fungicidal activity in a combination of amphotericin B, mainly targeting germ tube formation in *C. albicans* (Silva et al., 2011). According to the studies, *Rumex* root extract and Nepodin **88,** a compound extracted from *Rumex* roots, were defined as anti-biofilm agents against *C. albicans.* It also increased the survivability in nematode infection model. Further, Nepodin also showed repressed expression of several genes related to bud to hyphal transition Like *ECE1*, *UME6, HWP1*, and *HGT10*, while the increase in the expression of quite a few transport genes (*CDR4*, *TPO2*, and *CDR11*) essential for phenotypic expression (Lee et al., 2019). Additionally while screening compounds from the crude extract of aerial parts of *Waltheria indica*, 10 quinoline alkaloids were identified as a potential molecule which inhibit biofilm formation in *C. albicans*: These include Waltherione N **89**, (R)-Vanessine **90**, Waltherione Q **91**, 8-deoxoantidesmone **92**, Antidesmone **93**, Waltherione E **94**, Waltherione G **95**, Waltherione I **96**, Waltherione J **97**, Waltherione F **98** (Cretton et al., 2016; Table 6).

**TABLE 6:** Chemical structures and MIC/IC_50_ values of small molecule inhibitors (85–98).

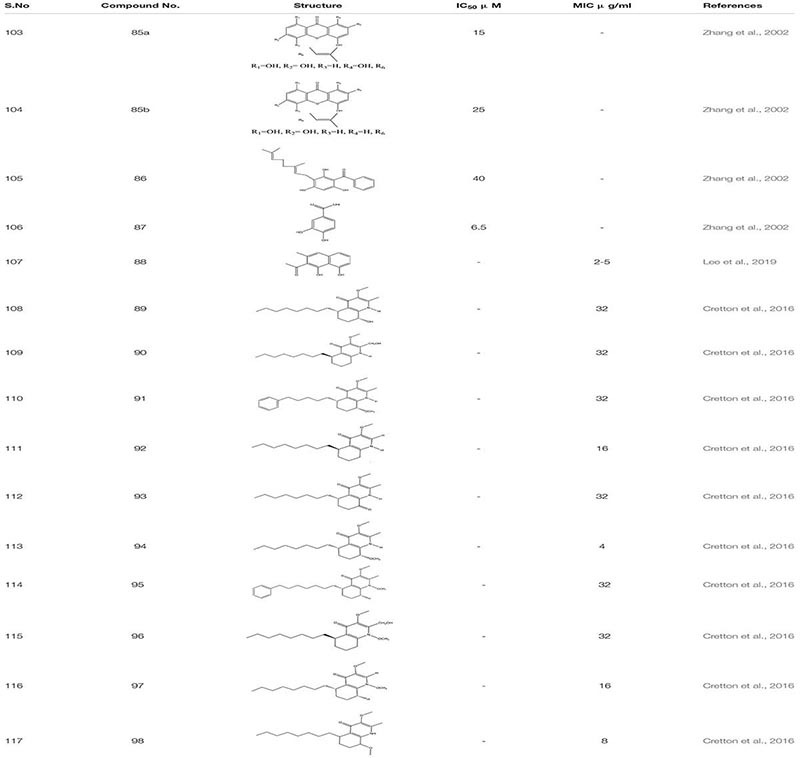

The Unexplored Brazilian Organic Propolis –BOP6 crude extract was characterized as an anti-inflammatory and anti-biofilm agent. It reduces the mortality rate by 30% in *C. albicans* infected sample with MIC ranging from 50 to 100 μg/mL while in combination with Amphotericin B, it decreases to 0.25 to 1.0 μg/mL (Nani et al., 2019). Through library screening with the information regarding fungal extract, it was revealed that *Bionectria ochroleuca*, an endophytic fungus. when cultivated on cereals, lead to the production of certain secondary metabolites-Polyketide glycosides **99**. These Polyketide glycosides exhibited anti-biofilm activity against *C. albicans* and correspondingly showed a synergistic effect with Amphotericin B (Wang et al., 2014).

From HPLC, a different set of compounds were extracted from the crude dichloromethane extract from the roots of *Swartzia simplex.* In total 14 compounds were identified out of which only six compounds such as Simplexene A **100**, (5S,10S)-11,15(R)-Dihydroxy,12 methoxyswartziarboreol G **101**, Simplexene B **102**, Simplexene D **103**, 11,12-Dihydroxy-15, 16-dihydroswartziarboreol C**104**,11,12-Dihydroxy-8,11,13,15- cassatetraen-17,16-olide(11,12 Dihydroxyswartziarboreol C) **105** depicted strong biofilm inhibition against *C. albicans* (Favre-Godal et al., 2015; Table 7).

**TABLE 7:** Chemical structures and MIC/IC_50_ values of small molecule inhibitors (99–105).

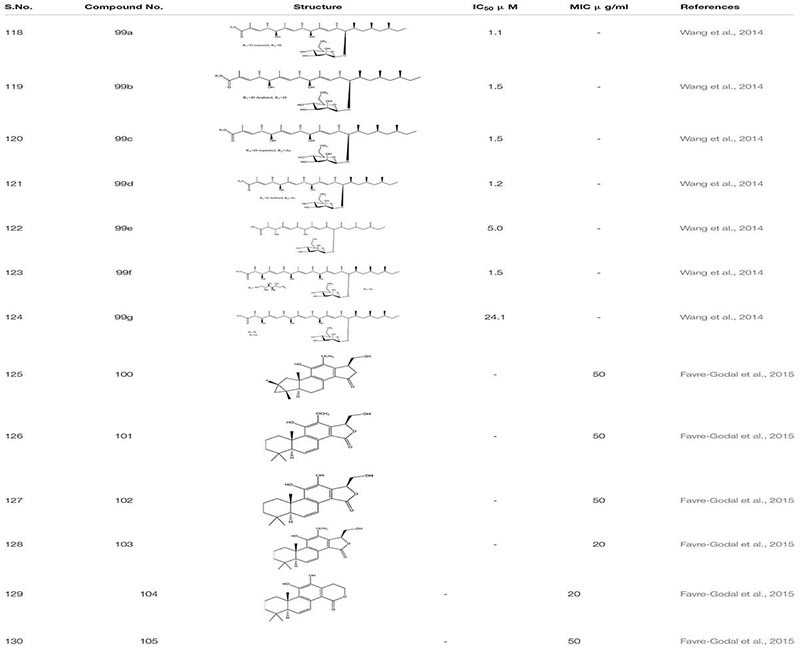

The crude extract of eucalyptus oil and its component 1, 8- cineole **106** showed antimicrobial activity against different microbes including *C. albicans* biofilm. It showed inhibitory effect alone and also in combination with chlorhexidine (Hendry et al., 2009; Simsek and Duman, 2017). While studying different components of essential oils as a treatment against invasive candidiasis, some terpenic derivatives showed promising results. These include carvacrol **73**, geraniol **75**, carvone **78**, terpinen-4-ol**107**, linalool **108**, menthol **109**, α-terpineol **110**, nerol **111**, isopulegol **112**, menthone **113,** and α-thujone **114.** On further study, tyrosol **115** –(a phenethyl alcohol), tyrosine derivative and eugenol- phenylpropanoid **116** both showed a strong inhibitory effect against *C. albicans* biofilm (Marcos-Arias et al., 2011; Raut et al., 2013). Furthermore, tyrosol **115** showed antibiofilm activity when applied exogenously at millimolar concentration. However, at micromolar concentration this compound stimulates hyphal production during the intial stage of *C. albicans* biofilm formation. This inhibition of biofilm formation by the exogenous addition of quorum sensing molecule usually occurs due to disruption in the qurom sensing mechanisms or due to the limitation of yeast adhesion on medical devices (Monteiro et al., 2015; Sebaa et al., 2019).

Epigallocatechin-3-gallate **117**-a Polyphenol compound from green tea extract and its analogs depicted a 75% reduction in the mature *C. albicans* biofilm (Evensen and Braun, 2009).

The α-Longipinene **118,** a significant component of *Helichrysum* oil and *Cascarilla* bark oil, inhibited the *C*. *albicans* biofilm formation without affecting planktonic cell growth and also exhibited a decrease in the virulence in *C. elegans* model (Manoharan et al., 2017a). Further, the compounds Glabridin **119**, Licochalcone A **120**, and Glycyrrhizic acid **121** derived from Licorice- a natural compound extracted from the root of the plant *Glycyrrhiza glabra* showed biofilm inhibition against *C. albicans.* It was found that Glabridin, Licochalcone A showed 35-60% of biofilm inhibition while they inhabited more than 80% hyphal growth. Furthermore, they displayed a synergistic effect with Nystatin at sub-inhibitory concentration (Messier and Grenier, 2011). Another successful study on the essential oils obtained from Thyme species – *T. camphoratus* and *T. carnosus*, earlier used in Portuguese as an antimicrobial therapy, lead to the discovery of different molecules.

In *Thymus carnosus* oil, high aggregates of Camphene and Borneol were present while *Thymus camphoratus* oil *was rich in* 1,8-cineole and α-pinene. Both the oils were highly effective against *C. albicans* biofilm and germ tube formation with very little toxicity (Alves et al., 2019). Another category of essential oils *Syzygium aromaticum* and *Cymbopogon citratus*, largely used in Asia, especially in India, for the treatment of inflammation and skin infection, displayed a potent anti-biofilm activity against *C. albicans* strain (Sajjad et al., 2012). Furthermore, Safranal **122** and its thiosemicarbazone **122a** derivative, the bioactive compounds obtained from *Crocus sativa* when added together in *C. albicans* culture, acted as a potent inhibitor of biofilm and germ tube formation at 32 times less concentration than MIC (Carradori et al., 2016; Table 8).

**TABLE 8:** Chemical structures and MIC/IC_50_ values of small molecule inhibitors (106–122).

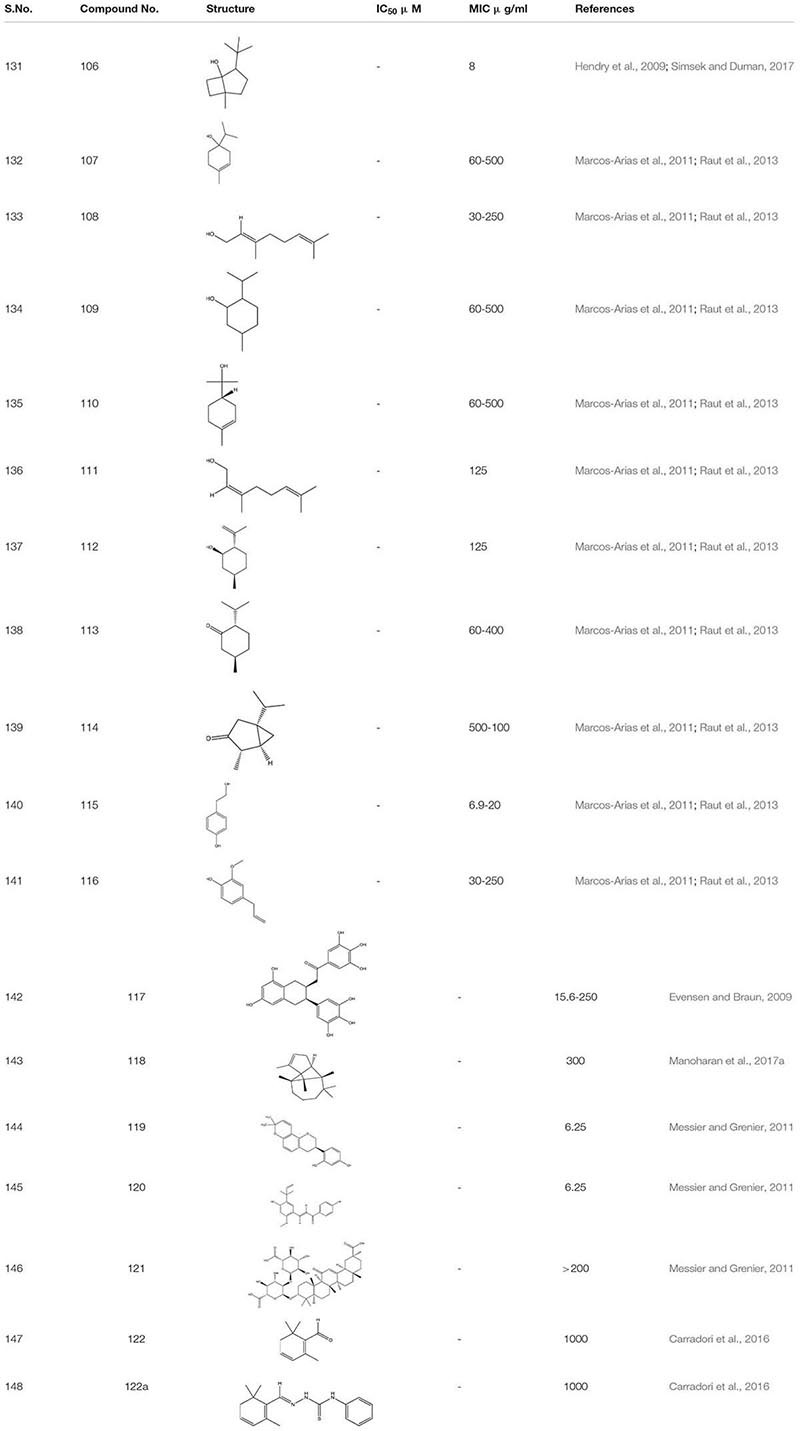

Work on small molecular inhibitors of plant origin, several molecules showed high to moderate activity toward *C. albicans* biofilm. These compounds like Cinnamaldehyde**123**, Piperidine **124**, Citral **125**, Furfuradehyde **126**, and Indole **127** showed inhibitory activity toward ergosterol biosynthesis at varying concentrations (Rajput and Karuppayil, 2013). A recent study revealed that compound **123** and **124** regulated antifungal activity against *C. albicans* by ROS mediated apoptosis (Chen et al., 2019; Thakre et al., 2020). Similarly, another set of *C. albicans* biofilm inhibitor Shearinines D and E **(128,129)** derived from *Penicillum species* showed yeast to hyphal blockage. However, it was observed that these compounds are more potent against early biofilm formation than the mature biofilm (You et al., 2013). Gymnemic acid **130,** a small molecular inhibitor obtained from the leaves of the plant *Gymnema sylvestre*, showed its activity against dual-species *S. gordonii* and *C. albicans* together, leading to biofilm formation in the mouth. It decreases the amount of e-DNA present in biofilm. It also inhibits the adhesive property of *C. albicans* by inhibiting an enzyme glyceraldehyde-3-phosphate dehydrogenase, which is thought to be responsible for the display of adhesion property in *C*. *albicans* (Veerapandian and Vediyappan, 2019). Similarly, Waikiloid A **131**- a prenylated indole alkaloid and polyketide metabolite Waikialide A**132** obtained from a Hawaiian *Aspergillus spp.* both acted as a potent biofilm inhibitor against *C. albicans* with a IC_50_ - 1.4 μM and 32.4 μM, respectively (Wang et al., 2012).

Myriocin **133** is a metabolite of the *Isaria sinclairii - a pathogenic insect* fungus. It disrupts the formation of sphingolipids as it is a structural analog of sphingosine precursors. It works very effectively in combination with known antifungal drugs like amphotericin and fluconazole, reducing the mortality rate from 26% to 0%, respectively. Additionally, *G.mellonella* larva infected with *C. albicans* showed a decrease in mortality rate when treated with myriocin. Furthermore, study of compound **133** indicated that it regulates *C. albicans* pathogenesis through NF-κB pathway, G protein coupled receptor and immunity (Melo et al., 2013). Acetylcholine **134,** a known soluble protein that has a neuronal function, also displays strong anti-biofilm activity against *C. albicans*. Along with this, it also reduces the damage induced due to inflammation in the host. Additionally, *in vivo* study of acetylcholine on *G. mellonella* larvae showed robust antifungal potency with a decrease in mortality rate from 80 to 100% to 25%. Further, compound **134** acts by promoting the rapid cellular immune response in host cells (Rajendran et al., 2015).

Garcinol **135** and Xanthochymol **136**, isoprenylated benzophenones obtained from *Garcinia xanthochymus* fruits, show antibiofilm activity against *C. albicans* by inducing programmed cell death in early biofilms without actually affecting the growth and viability of planktonic cells. Sampangine B **137, 137(a-b)** a naturally derived azaoxoporphine alkaloid and its derivatives showed potent activity against *C. albicans* biofilms and yeast to hyphal formation. It was found to show antifungal activity by inducing the accumulation of ROS and subsequent heme dysfunction. Moreover, it showed strong *in vivo* antifungal activity with low cytotoxicity in nematode model of *C. albicans* (Wu et al., 2017; Table 9).

**TABLE 9:** Chemical structures and MIC/IC_50_ values of small molecule inhibitors (123–137).

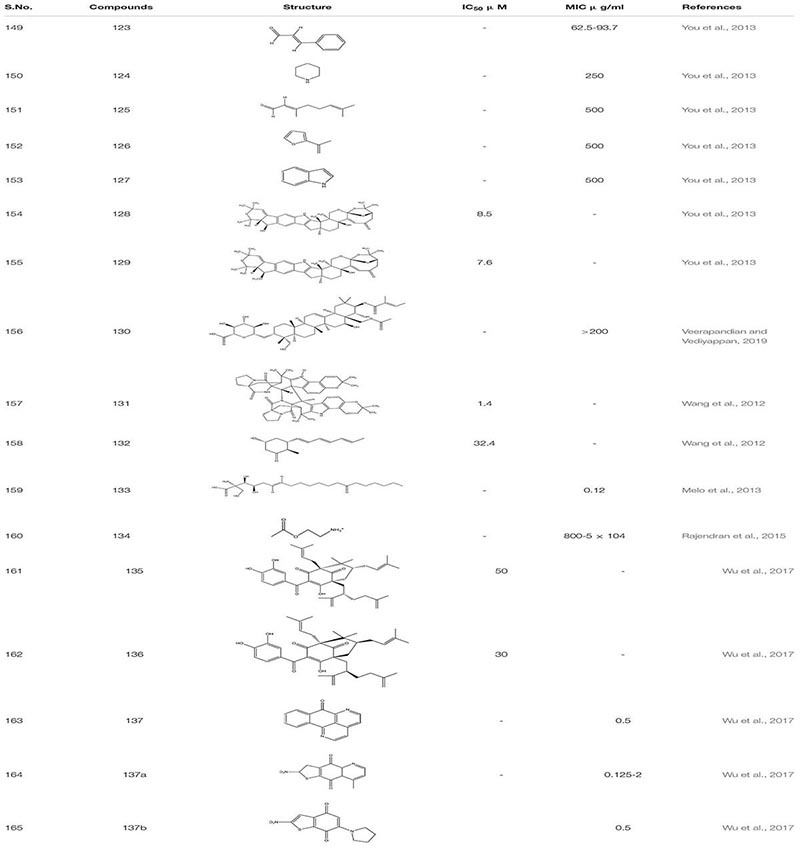

## Recent Advances to Inhibit *C. albicans* Biofilm Formation

Throughout this article, we have seen different strategies and methods to combat antifungal drug resistance. However, after all the efforts and attempts we are still stuck at a point where biofilm formation is difficult to treat with both non-antifungal and antifungal drugs because of the poor penetration and non-specificity of the drugs. Therefore, to address this issue, researchers are working on finding ways to increase the penetration of drugs into the extracellular matrix of biofilm. Over the last few years, different metal nanoparticles have emerged as prospective candidates to treat microbial infections pertaining to their strong potential as antimicrobial agents. In this context, different research groups have initiated the exploration to identify the antibiofilm activity of different nanomaterials against *C. albicans* (Shukla, 2020).

The usage of silver as an antimicrobial agent spans a century. Silver was used as a disinfectant by Greeks and Romans to decontaminate their water and food supplies. Silver was also used in ancient times to treat burns and wounds (Alexander, 2009; Barillo and Marx, 2014; Adhya et al., 2015). Studying silver nanoparticles against biofilms has gained immense recognition. Silver nanoparticles exert an inhibitory effect against fungal biofilms by damaging cell wall mostly by distortion and disruption of the outer surface of the fungal cell wall (Lara et al., 2015). In subsequent experiments, AgNPs when used to functionalize medical and environmental surfaces, demonstrated biofilm inhibition (>50%) at relatively low concentrations (2.3 to 0.28 ppm) (Lara et al., 2020). Combination therapy of Fluconazole with silver nanoparticles has also shown a substantial decrease in the MIC (Longhi et al., 2016). The resistance to fluconazole (FLC) in *C. albicans* is strongly associated with overexpression of genes encoding efflux pumps or lanosterol 14α-demethylase (Sagatova et al., 2016). Subsequently, with the disruption of the cell wall and cytoplasmic membrane of *C. albicans* by AgNPs, these silver particles cause an increase in reactive oxygen species and hydroxyl radical production, which can also contribute to cell membrane damage (Hwang et al., 2012; Monteiro et al., 2012). Recently, researchers have identified bismuth oxide (BiO_3_) nanoparticles with potent antimycotic activity against *C. albicans* growth. Moreover, when these nanoparticles were compared to commercially available antifungal drugs like chlorhexidine, nystatin, they completely eradicated the biofilm formation (Hernandez-Delgadillo et al., 2013). In another study, magnesium oxide nanoparticles (MgO NP) were used to investigate their antifungal and antibiofilm property against *C. albican*s. The study revealed that MgO nanoparticles effectively inhibited *C. albicans* biofilm formation. Furthermore, adhesion experiments showed that MgO NPs repressed the preliminary adhesion of *Candida albicans* (Kong et al., 2020). In another study, when gold nanoparticles (Au-NP) were tested on the biofilms formed due to *C. albicans*, these particles showed robust inhibitory activity. Additionally, these Au-NPs increased the host immune response activity against these pathogenic organisms (Yu et al., 2016; Nani et al., 2019). From previous studies, chitostan and its nanoparticle (ChNPs) were already known for their antifungal property (Ing et al., 2012; Mohammed et al., 2017; Gondim et al., 2018). Therefore, to establish its antibiofilm activity, ChNPs were investigated, and it was found that ChNPs significantly inhibited the biofilm formation causing very less changes in the acrylic resin surface (Gondim et al., 2018). Likewise, another study conducted with selenium nanoparticles (Se-NP) showed strong inhibition on *Candida* biofilm formation as these particles out compete sulfur in biological process due to their similar chemical properties. Also, these nanoparticles enter into the cell to shrink and disrupt the outer membrane structure of *C. albicans* cell (Guisbiers et al., 2017).

The titanium oxide nanoparticles (Ti-NP) when scrutinized for their antifungal property showed strong antifungal activity against planktonic form of *C. albicans.* They also depicted robust potential against biofilm formation (Haghighi et al., 2013). In addition, zinc oxide nanoparticles (ZnO-NPs) used on *Candida* biofilm showed a decrease in the biofilm formation by abot 62%-85% at varied concentration of 125 ppm to 250 ppm respectively. The ZnO-NPs suggested to inhibit hyphae formation in *C. albicans* by production of reactive oxygen species in a dose-dependent manner (Hosseini et al., 2018; Golipour et al., 2019; Mahamuni-Badiger et al., 2020). Additionally, another study involving copper oxide nanoparticles (Cu_2_ONP and CuONP) showed potent inhibitory action against biofilm formation by *C. albicans.* The study revealed that copper oxide nanoparticles constrain the yeast for hypae transformation. Also CuO-NP elicited the reactive oxygen species while Cu_2_ONP pronounced the membrane damage in *C. albicans.* In comparison to both types of copper oxide nanoparticles, CuONP was more stable and depicted better antifungal activity in comparison to Cu_2_O-NP (Rasool, 2019; Padmavathi et al., 2020). In another study, iron-oxide nanoparticles (Fe_3_O_4_ NPs) were investigated on *C. albicans* biofilm which showed 87.2-100% inhibition based on their particle size which was 100 ppm to 200 pm respectively (Seddighi et al., 2017).

The nitric oxide nanoparticles (NO-NP) showed strong potential as an antifungal agent by hindering the extracellular matrix and biofilm formation on the surface of biomaterials. It was found that NO-NPs also decrease the metabolic activity of *Candida* cells both *in vitro* and *in vivo* (Ahmadi et al., 2016).

## Concluding Remarks and Future Perspective

The high mortality and morbidity rate owing to *C. albicans* biofilm infection is a big challenge in medical mycology. Since the formation of biofilm and biomaterial infections is a progressively alarming problem, this warrants the development of new antifungal agents and the search for newer targets. This review elucidates the pathogenic foundation and molecular mechanism of *C. albicans* biofilms over antifungal drug resistance. A plethora of studies by several scientific groups and investigators have delivered essential knowledge regarding the pathogenesis associated with biofilm formation. These useful insights can represent an optimal starting point to find new therapeutic strategies related to drug resistance and mechanistic signals that govern *C. albicans* biofilm formation. Recent advances on different transcription factors, quorum sensing molecules, host response to adhesion, change in efflux pumps, enzymes, bud to hyphal transition, and change in lipid profile have broadened the knowledge of the complex mechanism underlying the biofilm resistance. Moreover, the development of new biomaterials with anti-adhesive properties, anti- infective lock therapies, high throughput phenotypic screening of small-molecule inhibitors, discovery and repurposing of naturally known compounds are under scrutiny. For medical instruments, a fine coating of nanomaterial may inhibit bacterial accumulation and biofilm formation. Recently, different metal nanoparticles have also emerged as antibiofilm agents against *C. albicans* and gaining momentum. Furthermore, different combinational therapies are harnessed for antibiofilm activity. Yet, the target-based approach is the need of the hour. For this multifaceted biofilm, the complex should be extensively studied to target different loops of phenotypic character change as they play a major role in biofilm formation. The detailed electron microscopic studies using both transmission electron microscopy (TEM) and scanning electron microscope (SEM) with much greater resolution may add additional repertoire to our knowledge. Yet another approach may be the use of some engineered enzymes that do not allow colony formation and inhibit in turn the biofilm formation. Any compound used for this purpose may be empirically optimized for its dose, sensitivity, and efficacy before a search for a target is mounted.

Given the diverse strategies to combat antifungal resistance, there is a hope that specific target-based drugs will be added to the arsenal in our fight against *C. albicans* biofilm formation in the not too distant future.

## Author Contributions

All authors contributed equally and approved the final version of the manuscript.

## Conflict of Interest

The authors declare that the research was conducted in the absence of any commercial or financial relationships that could be construed as a potential conflict of interest.
